# Reverse Offset Printed, Biocompatible Temperature Sensor Based on Dark Muscovado

**DOI:** 10.3390/s22228726

**Published:** 2022-11-11

**Authors:** Shahid Aziz, Junaid Ali, Krishna Singh Bhandari, Wenning Chen, Sijia Li, Dong Won Jung

**Affiliations:** 1 Department of Mechanical Engineering, Jeju National University, 102 Jejudaehakro, Jeju-si 63294, Korea; 2 Optoelectronics Research Laboratory (OERL), Department of Physics, COMSATS University Islamabad, Islamabad 45500, Pakistan

**Keywords:** reverse-offset printing, biocompatible, temperature sensor, sucrose, resistance, IDT

## Abstract

A reverse-offset printed temperature sensor based on interdigitated electrodes (IDTs) has been investigated in this study. Silver nanoparticles (AgNPs) were printed on a glass slide in an IDT pattern by reverse-offset printer. The sensing layer consisted of a sucrose film obtained by spin coating the sucrose solution on the IDTs. The temperature sensor demonstrated a negative temperature coefficient (NTC) with an exponential decrease in resistance as the temperature increased. This trend is the characteristic of a NTC thermistor. There is an overall change of ~2800 kΩ for the temperature change of 0 °C to 100 °C. The thermistor is based on a unique temperature sensor using a naturally occurring biocompatible material, i.e., sucrose. The active sensing material of the thermistor, i.e., sucrose used in the experiments was obtained from extract of Muscovado. Our temperature sensor has potential in the biomedical and food industries where environmentally friendly and biocompatible materials are more suitable for sensing accurately and reliably.

## 1. Introduction

The rapid advancement in modern day science and technology has initiated a huge demand for accurately measuring localized and surrounding environment temperatures. This has compelled the development of fascinating temperature sensors with novel nanomaterials, designs, structures, and sensing mechanisms to target the needs of measuring accurate temperatures in a variety of environments ranging from beyond ionosphere to inter-atomic and micro spaces between transepithelial/transendothelial animal cells. Monitoring and control of temperatures in an ambient atmosphere is important for our daily lives, industrial processes [1], health [2,3] and environmental [4] monitoring. The thermistor-based [5] temperature sensor is one of the most important candidates for temperature sensing that has outstanding advantages of immunity to electromagnetic interference, simple fabrication, cost-effectiveness, and durability against harsh environments. Thermo-resistive devices are extremely sensitive to the external temperature; therefore, by applying a thermo-resistive-based material, a temperature sensor with high sensitivity and practicability can be obtained [6].

Organic [7], bioinspired [8], biocompatible [9], and disposable [10] sensors have great potential for applications, such as in the food industry in the handling, processing [11], and storing of food items which relies on thermistors for safe and cost-effective operations. Likewise, these sensors are suitable for health monitoring [2,3], environmental monitoring of experimental labs [12] and medical industry [13]. Various types of temperature sensors have been fabricated, which include thermocouples [14], nanogenerator-based temperature sensors [15], and thermistors [16] etc.

Thermoplastic polymer composite containing metallic filler-based thermistors have been used as temperature sensors [17], which show linear responses from 20 to 200 °C. Polydiacetylene thermoresponsive fluorogenic supramolecules have been used as temperature sensor in microfluidic devices [18]. An extremely simple thermocouple made of a single layer of metal [19] has been fabricated by photolithography. A lead zirconate titanate (PZT) single micro/nanowire pyroelectric nanogenerator has been utilized as a self-powered temperature sensor [20]. A roll-to-roll compatible laser-induced carbon on Kapton reported a temperature sensitivity in sub-zero temperature ranges of 0 °C to −150 °C [21]. The ability of ZnO-Graphene composite was exploited to demonstrate a composite coated micro-nanofiber temperature sensor exhibiting high sensitivity in range of 25 °C to 60 °C. A porous graphene/polydimethylsiloxane sensor demonstrated sensitivity of 5%/°C for thermal sensitivity from 30 °C to 70 °C [22]. A gold doped silicon on ultrathin polymer layer in serpentine mesh structure demonstrated ultra-sensitivity and stretchability as epidermal temperature sensor array [23]. Gold nanoparticle-loaded temperature-responsive dendrimers, such as carboxy-terminal phenylalanine-modified polyamidoamine dendrimer, produce naked-eye colorimetric sensors for temperature-sensing applications [24].

In this paper, we present the study of thermoresistive effect in sucrose (C_12_H_22_O_11_) coated on IDTs on a glass substrate. The sucrose film is integrated on highly sensitive IDTs, whose resistance changes dramatically once the temperature changes.

## 2. Materials and Methods

### 2.1. Materials

The silver nanoparticles (AgNPs) ink were obtained from PARU Co., Ltd., Suncheon, South Korea. The AgNPs ink (Model: PG-007) had an average diameter of (20–100 nm), Ag contents of ~95% wt% and typical resistance of 3.5 mΩ/sq/mil. Sucrose used in the fabrication of sensing film was purchased from local superstore which had a natural unrefined dark Mucovado sugar with its origins from the African Island nation of Mauritius. The outer layer of the blanket roll was a PDMS blanket sheet obtained from RPE Co., Ltd., Paju, South Korea. The cliché substrate was manufactured from stainless-steel via photolithography to engrave a micropattern design by Handu Co., Ltd., Seoul, South Korea. All the solvents were purchased from Duksan pure Chemicals, Co., Ltd., Ansan, South Korea.

### 2.2. Experimental

#### 2.2.1. Sensor Fabrication

**Figure 1:**
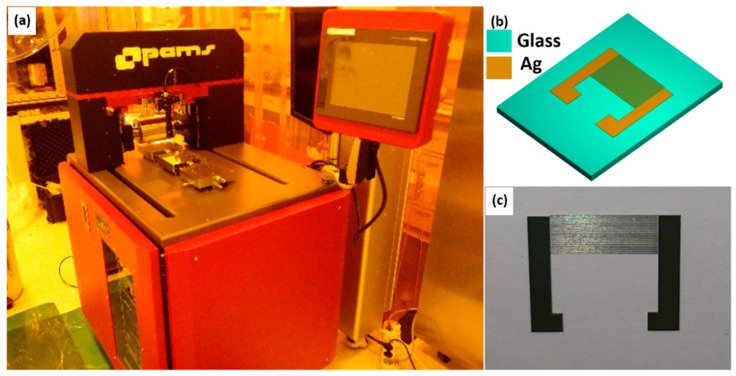
(**a**) Real photograph of reverse-offset printing system, (**b**) CAD design, and (**c**) real photograph of the Ag IDTs printed on glass.

The device fabrication started with printing [25] of Ag IDTs on glass substrate using reverse-offset printing. The Printed IDTs with 20 pair of IDTs are shown in Figure 1. The setup of semi-automatic reverse-offset printing system includes a glass slide to deposit ink, a roller enfolded with a blanket of highly hydrophobic poly-dimethylsiloxane (PDMS) film, a cliché imprinted with the negative image of IDT electrode patterns acutely carved in its surface in the form of trenches of rectangular cross-section and finally the chosen substrate for device fabrication.

**Figure 2:**
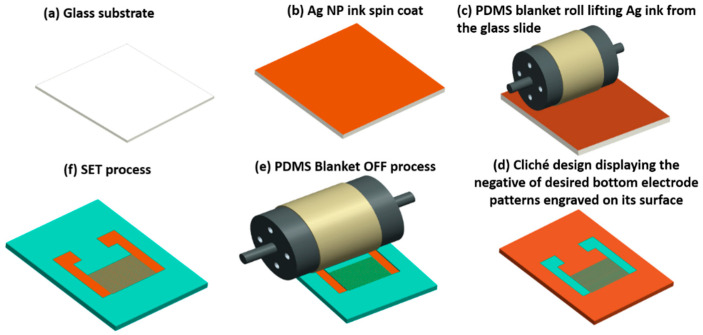
Schematic illustration of reverse-offset printing of Ag IDTs on glass substrate.

The printing of electrodes on glass substrate was conducted by following a series of predetermined steps. Firstly, the glass slide was sequentially cleaned with DI water, Ethanol, and acetone. In order to remove any remaining residuals from the surface of glass slide, it was treated with UV Ozone plasma. Once the substrate cleaning process was completed, a desired amount of silver ink was coated on the glass slide via a spin coater (ACE-200) at 3000 rpm. The silver coated glass slide can be seen in Figure 2b. As a subsequent step, as shown in Figure 2c, a cylindrical roll blanketed with a thick sheet of Polydimethylsiloxane (PDMS) was rolled over the silver coated glass slide. As a result, a thin uniform Ag film was peeled off form the glass surface and coated on the PDMS surface as it has a higher absorption coefficient than glass. To achieve the desired electrode patterns, a cliché of steel with engraved electrode patterns in the form of trenches was placed in the printing direction of the roll. The roll moved linearly on top of the cliché and then rolled over the entire cliché surface at an optimized speed and pressure. As a result, all the unwanted Ag ink from the PDMS roll was transferred to the cliché surface except from the regions moving over the engraved electrode patterns, causing the Ag ink to remain stuck to the PDMS roll exactly in the shape of desired electrode patterns. This process (lift-off process) can be seen in Figure 2d. It can be seen in the schematic figure that the unwanted silver ink (shown in dark brown color) has been transferred to the glass slide except for the engraved IDT patterns. As a final step, the PDMS blanket roll was rolled over the target glass substrate at an optimized speed and pressure to print the high-quality patterns as Ag IDT electrodes [26]. This process (set process) is shown in Figure 2e,f.

Very fine bottom Ag electrodes with 50 µm finger width resolution and outstanding resistivity of 0.4 ohm-cm were obtained after sintering for 1 h at 110 °C in a convection furnace.

The sucrose solution was synthesized by simply dissolving edible table sugar in DI water at room temperature. The equation for sucrose solution is as follows:

C_12_H_22_O_11_ (s) + H_2_O → (l) C_12_H_22_O_11_(aq) (R1)

First, 15 wt% of sucrose was dissolved in 10 mL of DI water in a glass bottle using a magnetic stirrer for 1 h at 50 °C and 1000 rpm. Once the Ag IDTs were sintered, they were coated by a thin film of sucrose solution (15 wt%) using a SCS 6800 spin coater at a speed of 1000 rpm. The devices were cured at 80 °C for 1 h to obtain sucrose coated IDTs on glass substrates. Finally, the connection wires were attached to each pair of IDTs through electrode pads (2 mm × 2 mm) using the silver epoxy and cured in a furnace at 100 °C for 10 min.

#### 2.2.2. Film Surface Morphology and Compositional Characterization

**Figure 3:**
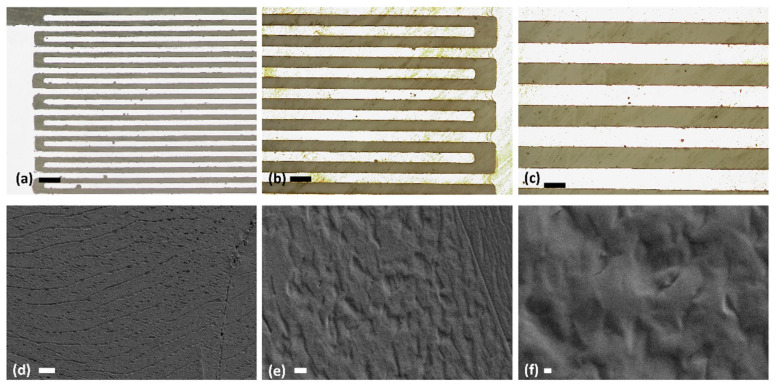
(**a**–**c**) Microscopic images of the reverse-offset printed IDTs after sintering, showing the dimensions of the IDTs fingers width and gaps, [scale bar- a: 200 µm, b: 100 µm, c: 50 µm] (**d**–**f**) SEM images of the sucrose film at different magnifications. Scale bars ((**d**): 10 µm, (**e**): 1 µm, (**f**): 200 nm).

The sensor structure consists of a pair of Ag IDTs (20 pairs of 50 µm × 20 mm each) coated with sucrose film. A stereo-zoom digital microscope (OLYMPUS CH30, Tokyo, Japan) was used to perform optical microscopy. The microscopic images of sintered Ag IDTs can be seen in Figure 3a–c at the magnifications of 10×, 50× and 100×, respectively.

**Figure 4:**
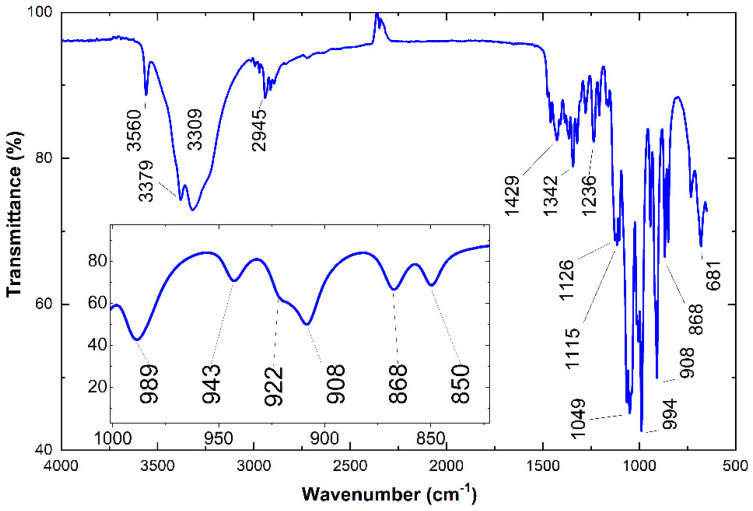
The FTIR spectra of sucrose. Inset shows the zoomed-in peaks in the region (650–1100 cm^−1^).

The images show a successful fabrication and sintering of Ag IDTs using the reverse-offset fabrication system. The width of each IDT finger and the gap between any two adjacent fingers of printed IDTs was perfectly aligned and maintained to be 50 µm. The surface morphology of the sucrose film was investigated by FESEM images using Carl Zeiss Supra 55VP. Figure 3d–f show FESEM images of the sucrose film at low and high resolutions indicating its highly uniform nature over the target substrate. Figure 4 shows the FTIR spectrum of the sucrose film sample. The data was recorded from 650 cm^−1^ to 4000 cm^−1^ in increments of 10 cm^−1^ at log(R^−1^), in which R represents the ratio of the reflected intensity of the IR from the background to that of the sample. Four different peaks were observed at 833 cm^−1^, 865 cm^−1^, 920 cm^−1^, and 996 cm^−1^. The signature peaks of sucrose as obtained from the absorption spectrum are in accordance with the literature [27,28].

The spectral region of our interest lies between 850 and 1100 cm^−1^, which has been shown in the inset of Figure 4 and in Table 1. This specific region is the characteristic absorption band of saccharides. This compositional analysis suggests that the chemical structure of sucrose (C_12_H_22_O_11_), which is a disaccharide, used to form the sensing film did not change after its treatment with DI water and heating at different temperatures (during curing and testing).

Sessile drop testing has been used for water contact angle θ_WCA_ measurements on a software with a drop shape analysis software [29]. Three measurements were analyzed for the films fabricated from three different concentrations of sucrose (5 wt%, 10 wt% and 15 wt%).

**Figure 5:**
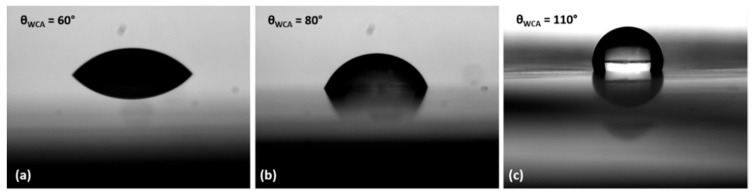
The photographs of water droplet on the sucrose coated surfaces with concentrations of 5 wt% (**a**), 10 wt% (**b**), and 15 wt% (**c**).

Figure 5 shows the contact angle measurement results. As the concentration of sucrose is increased, the resulting film after curing shows more hydrophobicity as compared to the lower concentrations of sucrose. Therefore, we used 15 wt% solution to fabricate the sucrose film as temperature sensing layer. This ensures a negligible change in the response of the sensor at changing humidity levels [30,31].

#### 2.2.3. Electrical Characterization

**Figure 6:**
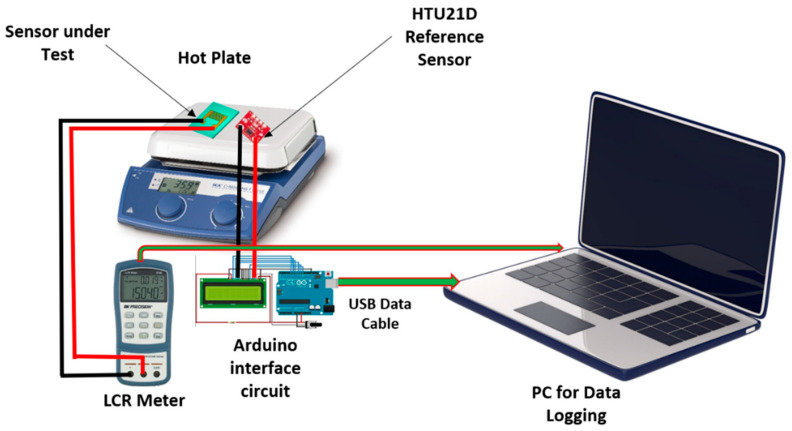
In-house built characterization setup for electrical characterization of temperature sensor.

The fabricated temperature sensor was tested for its electrical response towards temperature changes in a controlled temperature environment. The inhouse built characterization setup can be seen in Figure 6. The measurement setup consists of a hotplate to control the temperature, sensor under test, HTU21D reference sensor, LCR meter to obtain and display the electrical signals from the sensor being tested, Arduino interface circuit with LCD to display the real time readings of the temperature on the hotplate surface being recorded by the HTU21D reference sensor, USB data cables and a laptop for automatic data logging from the Arduino circuit and LCR meter.

An automatic feedback controller was used to control the temperature changes on the surface of the hotplate, whereas a manually controlled user input was used to change the temperature of the hotplate in steps of 5 °C per step. Once a step change of 5 degrees was done on the hotplate, the temperature was maintained at that point until the resistance readings of the sensors became stable. This was necessary to compensate for any differences in response times of the fabricated sensor and the HTU21D reference sensor and to overcome the hysteresis effect due to the material of the sensor. The sensors’ readings were recorded against the range of 0 °C to 100 °C while keeping the relative humidity (RH) of the controlled environment maintained at ~60% RH. As the temperature of the hotplate surface was increased or decreased through the controller, the consequent responses recorded by both sensors placed on the hotplate surface were recorded in the form of resistance (R) variations and the trends followed by the fabricated sensor were analyzed by plotting the curves in Origin Lab. [31,32].

## 3. Results and Discussion

The fabricated sensor is a type of negative temperature coefficient (NTC) thermistor and is mostly suitable for small volume, high accuracy, short response time, and its capability to be mass-produced. NTC thermistor’s resistance decreases exponentially with the increase in temperature. When the temperature of the sensing material is increased, the valence electrons at the molecular level become more active producing a negative temperature coefficient, i.e., they have a negative electrical resistance versus temperature (*R*/*T*) relationship. This decrease in resistance can be represented by the following equations:

(1) $$\beta =\frac{T_{t}\times T_{i}}{T_{i}-T_{t}}\times \ln\left(\frac{R_{i}}{R_{t}}\right)$$

In Equation (1), Δ*T* is change in temperature, *R_t_* and *R_i_* are the resistances of the thermistor at *T_t_* °C and *T_i_* °C and *β* is the temperature coefficient of thermistor. Equation (1) can be written as

(2) $$R=R_{i}\;e^{\beta \left[\frac{1}{T_{t}}-\frac{1}{T_{i}}\right]}$$

**Figure 7:**
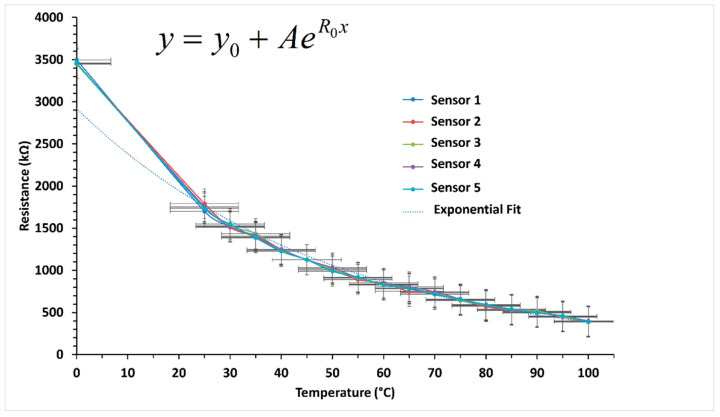
Temperature response of sensors in the form of resistance in kΩ as a function of temperature in °C.

Figure 7 shows the trend in response of the sensors results for five similarly fabricated sensors. As the temperature increases, the overall resistance of the sensor decreases according to the above equation. By using the Boltzmann second order curve fitting equations, *R*^2^ value ≥ 0.99 was achieved. This shows the possibility of easy conversion of sensor response to temperature in °C with quite high accuracy by solving the generated equation.

**Figure 8:**
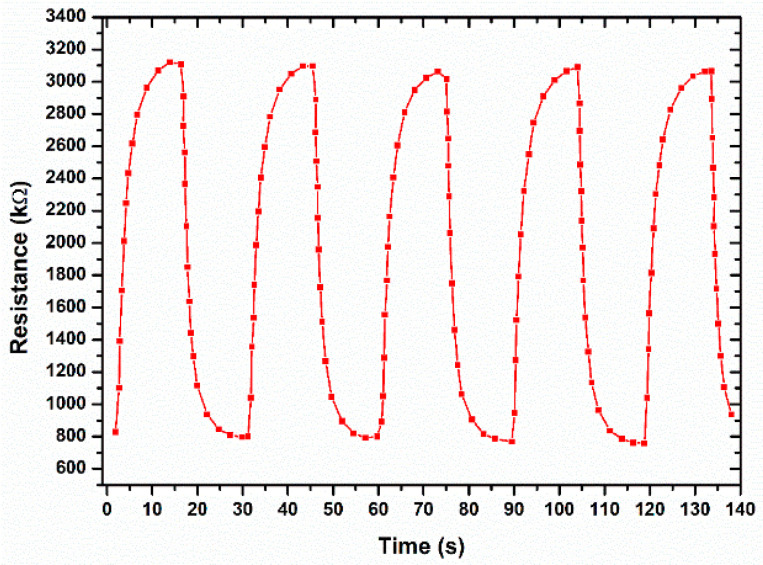
Response time of the temperature sensor at heating and cooling cycles.

The fabricated temperature sensor was subjected to five consecutive heating and cooling cycles to calculate the response and recovery time of our temperature sensor. The results have been plotted in Figure 8. The response time is the time taken by the sensor to achieve the value from 10% to 90% of the maximum value and the recovery time is the time taken to achieve the value from 90% to 10% of the maximum value. The response and recovery time values are average of the values taken from all the heating and cooling cycles. It can be observed that the response time of the temperature sensor is approximately 4 s, and the recovery time is nearly 6 s. The sensor output response shows quick response and recovery times which are characteristics of a good performance.

## 4. Conclusions

Naturally occurring materials are very important for environmentally friendly electronic devices such as sensors. Dark Muscovado-extracted sucrose has been utilized as a sensing material to fabricate a temperature sensor. The sensor has a simple two-layered structure with silver nanoparticle IDTs printed on a glass substrate and covered by a spin-coated sensing film of sucrose. The sensor electrodes (IDTs) were fabricated using a high precision reverse-offset printer to ensure high resolution printing at micron-level accuracy. The primary role of sucrose over resistance of the sensor by the temperature changes has been confirmed. The temperature sensor demonstrated the characteristics of a typical thermistor with negative temperature coefficient (NTC) while maintaining stable readings of resistance ranging from 400 kΩ to 3200 kΩ for a temperature range of 0 °C to 100 °C. The sensor has potential applications in the food and medical industries where stable temperature sensing is required in a wide temperature range.

## Figures and Tables

**Table 1:** Absorption peak signatures of FTIR spectroscopy of sucrose as indicated by Figure 4.

Peak Position (cm^−1^)	Reported Peak Positions (cm^−1^)	Position Error (cm^−1^)
833	834	1
865	869	4
920	924	4
996	994	2

## Data Availability

Not applicable.
